# Frequency Domain Repercussions of Instantaneous Granger Causality

**DOI:** 10.3390/e23081037

**Published:** 2021-08-12

**Authors:** Luiz A. Baccalá, Koichi Sameshima

**Affiliations:** 1 Departamento de Telecomunicações e Controle, Escola Politécnica, Universidade de São Paulo, São Paulo 05508-900, Brazil; 2 Departamento de Radiologia e Oncologia, Faculdade de Medicina, Universidade de São Paulo, São Paulo 01246-903, Brazil; ksameshi@usp.br

**Keywords:** instantaneous Granger causality, total partial directed coherence, information partial directed coherence, total directed transfer function, information directed transfer function, Granger connectivity, Granger influentiability

## Abstract

Using directed transfer function (DTF) and partial directed coherence (PDC) in the information version, this paper extends the theoretical framework to incorporate the instantaneous Granger causality (iGC) frequency domain description into a single unified perspective. We show that standard vector autoregressive models allow portraying iGC’s repercussions associated with Granger connectivity, where interactions mediated without delay between time series can be easily detected.

## 1. Introduction

Recent years have seen an abundance of approaches aimed at describing the ‘connectivity’ between sets of observed time series. To this end, *Granger causality*-based ideas [1] stand out prominently and involve a wide variety of time series techniques comprising time [1,2] and frequency domain [3,4,5] descriptions.

Granger causality descriptions are centered on determining how helpful the past of a time series can be insofar as predicting another time series. As such, eventual simultaneous relationships are not taken into account. This aspect is described via the so-called *instantaneous* Granger causality (iGC), which is deemed to be present whenever modeling residues between different time series are correlated.

This latter aspect has received relatively far less attention and, for a long time, this time series residue connection meant that the idea remained restricted to time domain considerations. This state of affairs was changed following the work of Faes and Nollo [6,7], who proposed adding extra coefficients to model interactions that are not mediated by delay from which such descriptors as *directed transfer function* (DTF) [3] and *partial directed coherence* (PDC) [4] could be generalized. More recently, an alternative [8] based on comparing models over suppressed time series has surfaced.

Rather than contrasting the latter descriptions, here, we wish to show that the formalism behind DTF and PDC can be naturally extended to include a reasonable frequency domain description of instantaneous Granger effects when their information versions iDTF/iPDC [9] are considered without the need for employing modified models as required by the other previous approaches [6,7,8].

This development is interesting furthermore since it also allows a rounded closed form description, not previously available for directed frequency domain relationships, that can be deduced from second order statistical information alone.

In the developments that follow, we shall employ the concepts of *Granger connectivity* (G–C) and *Granger influentiability* (G–I), introduced in [10], which refer respectively to PDC- and DTF-based descriptions of the ties between time series. The first one focuses on immediate connections between time series as opposed to the second one, which summarizes all possible signal pathways that join them. More information is available in [11].

The paper is organized as follows. Section 2 describes the main results after a brief recap of the essential concepts (Section 2.1), including how to write coherency and partial coherency in terms of iDTF/iPDC. This is followed by the newly proposed quantities of the *total directed transfer function* (tDTF) and *total partial directed coherence* (tPDC) in Section 3. Section 4 contains some brief numerical examples to illustrate the new concepts followed by a brief discussion (Section 5) and the ensuing conclusions (Section 6).

## 2. Problem Formulation

### 2.1. Preliminaries

We assume that the multivariate time series data $x\left(n\right)={\left[x_{1}\left(n\right),\ldots ,x_{N}\left(n\right)\right]}^{T}$ is adequately represented by the vector autoregressive model as follows:

(1) $$x\left(n\right)=\sum_{r}A_{r}x\left(n-r\right)+w\left(n\right),\;r>0$$

where $w\left(n\right)={\left[w_{1}\left(n\right),\ldots ,w_{N}\left(n\right)\right]}^{T}$ stand for zero mean innovation (white) processes with $\Sigma_{w}$ as its covariance matrix.

Instantaneous Granger causality corresponds to a non diagonal $\Sigma_{w}$.

Under these conditions, it is possible to describe the joint spectral matrix of x(n) as follows:

(2) $$S\left(\nu \right)=H\left(\nu \right)\Sigma_{w}H^{H}\;\left(\nu \right),$$

where ${}^{H}$ is the Hermitian transpose and

(3) $$H\left(\nu \right)={\bar{A}}^{-1}\;\left(\nu \right)$$

for $\bar{A}\left(\nu \right)$ defined as a matrix whose elements equal

(4) $${\bar{A}}_{ij}\left(\nu \right)=\left\{\begin{array}{c} 1-\sum_{r}a_{ij}\left(r\right)e^{-j2\pi \nu r},\;\mathrm{if}\;\;i=j\\ \;\;\;-\sum_{r}a_{ij}\left(r\right)e^{-j2\pi \nu r},\;\mathrm{otherwise} \end{array}\right.$$

with $j=\sqrt{-1}$.

The elements of (2) are thus given by

(5) $$S_{ij}\left(\nu \right)=h_{i}\left(\nu \right)\Sigma_{w}h_{j}^{H}\;\left(\nu \right)$$

where $h_{k}\left(\nu \right)=\left[H_{k1}\left(\nu \right),\ldots ,H_{kN}\left(\nu \right)\right]$ is the *k*-th row of H(ν).

This immediately leads to the coherency between $x_{i}\left(n\right)$ and $x_{j}\left(n\right)$:

(6) $$C_{ij}\left(\nu \right)=\frac{S_{ij}\left(\nu \right)}{\sqrt{S_{ii}\left(\nu \right)\;S_{jj}\left(\nu \right)}}=\frac{h_{i}\left(\nu \right)\Sigma_{w}h_{j}^{H}\;\left(\nu \right)}{\sqrt{\left(h_{i}\left(\nu \right)\Sigma_{w}h_{i}^{H}\;\left(\nu \right)\right)\left(h_{j}\left(\nu \right)\Sigma_{w}h_{j}^{H}\;\left(\nu \right)\right)}}.$$

In [9] we defined *information directed transfer function* (iDTF) as

(7) $$\gamma_{ij}\left(\nu \right)=\frac{\sigma_{j}H_{ij}\left(\nu \right)}{\sqrt{h_{i}\left(\nu \right)\Sigma_{w}h_{i}^{H}\;\left(\nu \right)}}.$$

Hence, we can express (6) as

(8) $$C_{ij}\left(\nu \right)=\gamma_{i}\left(\nu \right)\;R\;\gamma_{j}^{H}\;\left(\nu \right),$$

where the R matrix collects the $\rho_{ij}$ correlation coefficients between $w_{i}\left(n\right)$ and $w_{j}\left(n\right)$ and where $\gamma_{k}\left(\nu \right)=\left[\gamma_{k1}\left(\nu \right),\ldots ,\gamma_{kN}\left(\nu \right)\right]$ stands for the *k*-th row of what we define as the iDTF matrix Γ(ν). One might collect the quantities in (8) as elements of the *coherency matrix* as follows:

(9) $$C\left(\nu \right)=\Gamma \left(\nu \right)\;R\;\Gamma^{H}\;\left(\nu \right)$$

The instantaneous Granger causality is absent if and only if R reduces to the N×N identity matrix $I_{N}$.

In [12] we showed that the *partial coherency* between pairs of time series $x_{i}\left(n\right)$ and $x_{j}\left(n\right)$ within the x(n) set can be written as

(10) $$\kappa_{ij}\left(\nu \right)=\frac{{\bar{a}}_{i}^{H}\;\left(\nu \right)\Sigma_{w}^{-1}{\bar{a}}_{j}\left(\nu \right)}{\sqrt{\left({\bar{a}}_{i}^{H}\;\left(\nu \right)\Sigma_{w}^{-1}{\bar{a}}_{i}\left(\nu \right)\right)\left({\bar{a}}_{j}^{H}\;\left(\nu \right)\Sigma_{w}^{-1}{\bar{a}}_{j}\left(\nu \right)\right)}},$$

where ${\bar{a}}_{k}\left(\nu \right)$ stands for the *k*-th column of $\bar{A}\left(\nu \right)$.

For convenience, let D be a diagonal matrix collecting the standard deviations $\sigma_{i}$ from $w_{i}\left(n\right)$ so that

(11) $$\Sigma_{w}=D\;R\;D.$$

This implies that

(12) $$\Sigma_{w}^{-1}=D^{-1}R^{-1}D^{-1}$$

where one may further write

(13) $$R^{-1}=\bar{D}\;\tilde{R}\;\bar{D}$$

where $\bar{D}$ is a diagonal matrix with ${\tilde{\sigma }}_{i}$ elements that further reduce $\tilde{R}$ to a matrix of partial correlations ${\tilde{\rho }}_{ij}$ which is symmetric to those along the main diagonal.

If we rescale *information partial directed coherence* [9],

(14) $$\pi_{ij}\left(\nu \right)=\frac{{\bar{A}}_{ij}\left(\nu \right)/\sigma_{i}}{\sqrt{{\bar{a}}_{j}^{H}\;\left(\nu \right)\Sigma_{w}^{-1}{\bar{a}}_{j}\left(\nu \right)}},$$

as

(15) $${\bar{\pi }}_{ij}\left(\nu \right)={\tilde{\sigma }}_{i}\pi_{ij}\left(\nu \right),$$

then we can rewrite (10) as

(16) $$\kappa_{ij}\left(\nu \right)={\bar{\pi }}_{i}^{H}\;\left(\nu \right)\;\tilde{R}\;{\bar{\pi }}_{j}\left(\nu \right)$$

in complete analogy to (8), where ${\bar{\pi }}_{k}\left(\nu \right)={\left[{\bar{\pi }}_{1k}\left(\nu \right),\ldots ,{\bar{\pi }}_{Nk}\left(\nu \right)\right]}^{T}$ is the *k*-th column of what we name the iPDC matrix Π(ν), which allows writing the *partial coherency matrix* as

(17) $$K\left(\nu \right)=\Pi^{H}\;\left(\nu \right)\;\tilde{R}\;\Pi \left(\nu \right)$$

The rescaling (15) is what allows writing (8) and (16) in formally similar ways.


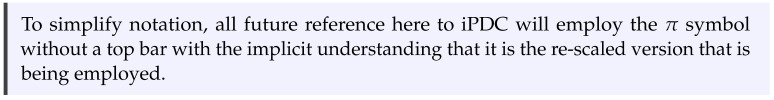


As before, it is easy to show that instantaneous Granger causality is absent if and only if $\tilde{R}$ reduces to $I_{N}$.

## 3. Total DTF and Total PDC

Before introducing the new quantities, some comments are due.

First of all, Equations (9) and (17) confirm the roles of iDTF and iPDC as factors of coherency and partial coherency as we have repeatedly stated [4], where the standard plots for them are organized as graph panels with the same layout, portraying the magnitude squared values of the entries in Γ and Π, respectively.

The originally defined directed transfer function [3] and partial directed coherence [4] are simplified forms of (7) and (14), respectively, by fully dispensing with the instantaneous aspects by replacing $\Sigma_{w}$ with $I_{N}$. Directed coherence [13] and generalized PDC (gPDC) [5] lend scale invariance to the latter quantities by replacing $\Sigma_{w}$ with a matrix comprised only of its diagonal elements in (7)/(14). This means that the latter forms do not suffer contamination from instantaneous effects as opposed to iDTF/iPDC which contain the full $\Sigma_{w}$ matrix in their definitions.

A couple of things are easy to show regarding DTF (DC)/PDC (gPDC). The first one is that, when N=2, $|DTF_{ij}{\left(\nu \right)|}^{2}={\left|PDC_{ij}\left(\nu \right)\right|}^{2}$ ($|DC_{ij}{\left(\nu \right)|}^{2}={\left|gPDC_{ij}\left(\nu \right)\right|}^{2}$) and $|DTF_{ii}{\left(\nu \right)|}^{2}={\left|PDC_{jj}\left(\nu \right)\right|}^{2}$ ($|DC_{ii}{\left(\nu \right)|}^{2}={\left|gPDC_{jj}\left(\nu \right)\right|}^{2}$). It is easy to show that the same properties hold between iDTF and the rescaled version of iPDC (15).

The second one is that fixing the target structure and adding the DTF/DC magnitude squared contributions from all sources adds to 1. A similar result holds for PDC/gPDC, except that now, one must fix the source and sum over the magnitude squared target structures.

However, even though at first sight, a strict normalization does not encompass iDTF or iPDC, one may show a similar property by noticing that

(18) $$C_{ii}\left(\nu \right)=1=\gamma_{i}\left(\nu \right)\;R\;\gamma_{i}^{H}\;\left(\nu \right)={\tilde{\gamma }}_{i}\left(\nu \right)\gamma_{i}^{H}\;\left(\nu \right)$$

and that

(19) $$\kappa_{ii}\left(\nu \right)=1=\pi_{i}^{H}\;\left(\nu \right)\;\tilde{R}\;\pi_{i}\left(\nu \right)=\pi_{i}^{H}\;\left(\nu \right){\tilde{\pi }}_{i}\left(\nu \right)$$

for

(20) $${\tilde{\gamma }}_{i}\left(\nu \right)=\gamma_{i}\left(\nu \right)R$$

(21) $${\tilde{\pi }}_{i}\left(\nu \right)=\tilde{R}\pi_{i}\left(\nu \right)$$

so that indeed it is the latter terms that lead to a normalization that reduces to that of DTF(DC)/PDC(gPDC) when $\Sigma_{w}$ is suitably replaced.

For future reference, we define ${\tilde{\gamma }}_{ij}\left(\nu \right)$ and ${\tilde{\pi }}_{ij}\left(\nu \right)$ of (20) and (21) as the $x_{j}\left(n\right)$ to $x_{i}\left(n\right)$*latent directed instantaneous* influentiability and connectivity, respectively. They represent would-be frequency domain repercussions due to instantaneous Granger causality when their respective j→i iDTF or iPDC are not zero.

Finally, one should note that, even though iDTF and iPDC have interpretations of their own in terms of mutual information rates between processes that describe the multivariate x(n) process [9], the fuller impact of the presence of instantaneous Granger causality is, however, mostly concentrated at the correlation R and partial correlation $\tilde{R}$ coefficient matrices.

We can write down all terms whose addition produce the various $C_{ii}\left(\nu \right)$ along the rows of a single matrix:

(22) $$\Gamma \left(\nu \right)\;R⊙\Gamma^{*}\;\left(\nu \right)$$

where ⊙ is Hadamard’s element-wise product, and ${}^{*}$ stands for complex conjugation.

However,

(23) $$R=I_{N}+\rho$$

where ρ stands for a matrix containing correlation coefficients as off-diagonal terms and whose main diagonal has only zeros.

Therefore, we may rewrite (22) as

(24) $$\Gamma \left(\nu \right)⊙\Gamma^{*}\;\left(\nu \right)+\Gamma \left(\nu \right)\rho ⊙\Gamma^{*}\;\left(\nu \right)$$

whose first term is readily recognizable as a matrix whose elements contain the magnitude squared of iDTF in the standard form. The second term isolates influences associated with iGC. Whereas the elements of the first term are real non-negative, the entries of the second term are inherently complex.

We propose to call (24) *total DTF* and denote it as

(25) $$\underset{\underset{\begin{array}{c} \mathrm{total}\;\mathrm{DTF} \end{array}}{︸}}{\overset{⏜}{\;\;\Gamma \;\;}\;(\nu )}=\underset{\begin{array}{c} \mathrm{Squared}\;\mathrm{iDTF} \end{array}}{\underset{︸}{\Gamma \left(\nu \right)⊙\Gamma^{*}\;\left(\nu \right)}}\;+\;\underset{\begin{array}{c} \mathrm{Residual}\;\mathrm{directed}\;\mathrm{DTF} \end{array}}{\underset{︸}{\Gamma \left(\nu \right)\rho ⊙\Gamma^{*}\;\left(\nu \right)}}$$

where its first term contains the customary **Granger influentiability** description [10] and the second its **directed instantaneous influentiability** counterpart. Both $\overset{⏜}{\;\;\Gamma \;\;}\;(\nu )$ and $\Gamma \left(\nu \right)\rho ⊙\Gamma^{*}\;\left(\nu \right)$ are complex quantities.

Clearly, the row elements of (25) sum to 1. Because the elements in the rows of $\Gamma \left(\nu \right)⊙\Gamma^{*}\;\left(\nu \right)$ are all real and non-negative, the sum of $\Gamma \left(\nu \right)\rho ⊙\Gamma^{*}\;\left(\nu \right)$ along a row is also a real number.

Since we can write

(26) $$\tilde{R}=I_{N}+\tilde{\rho }$$

we may define *total PDC* as

(27) $$\underset{\begin{array}{c} \mathrm{total}\;\mathrm{PDC}\\ \; \end{array}}{\underset{︸}{\overset{⏜}{\;\;\Pi \;\;}\;(\nu )}}=\underset{\begin{array}{c} \mathrm{Squared}\;\mathrm{iPDC} \end{array}}{\underset{︸}{\Pi^{*}\;\left(\nu \right)⊙\Pi \left(\nu \right)}}+\underset{\begin{array}{c} \mathrm{Residual}\;\mathrm{directed}\;\mathrm{PDC} \end{array}}{\underset{︸}{\Pi^{*}\;\left(\nu \right)⊙\tilde{\rho }\Pi \left(\nu \right)}}$$

where the entries in $\Pi^{*}\;\left(\nu \right)⊙\Pi \left(\nu \right)$ describe what we called **Granger connectivity** [10] and $\Pi^{*}\;\left(\nu \right)⊙\tilde{\rho }\Pi \left(\nu \right)$ its **directed instantaneous connectivity** counterpart.

The column-wise sum of the elements of (27) adds to one, whereas those of the columns of $\Pi^{*}\;\left(\nu \right)⊙\tilde{\rho }\Pi \left(\nu \right)$ sum to a real number since the elements of $\Pi^{*}\;\left(\nu \right)⊙\Pi \left(\nu \right)$ are non-negative real.

To facilitate reference, the key symbols are given in Table 1.

## 4. Numerical Examples

To provide some intuition, we examine the following numerical examples.

**Figure 1:**
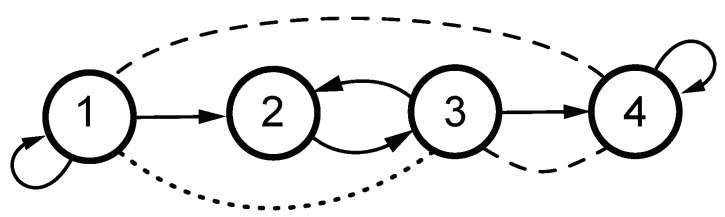
Link structure for the Example 1. Dashed lines indicate non zero covariance in $\Sigma_{w}$. The dotted line portrays the partial correlation aspect in (30).

**Example** **1.** *Consider a system whose connections are contained in Figure 1. Dashed lines represent instantaneous interaction aspects, while dotted lines reflect the additional instantaneous interaction aspect that becomes explicit upon $\tilde{R}$ computation (Equation (30)). The underlying system is a first order one given by (1) and defined by*

(28) $$A_{1}=[\begin{array}{llll} \;\;0.5 & 0 & \;\;0 & \;\;0\\ 1 & 0 & -0.95 & \;\;0\\ 0 & 0.95 & \;\;0 & \;\;0\\ 0 & 0 & \;\;1 & -0.75 \end{array}]$$

*with*

(29) $$\Sigma_{w}=[\begin{array}{llll} 1 & 0 & \;\;0 & \;\;0.25\\ 0 & 1 & \;\;0 & \;\;0\\ 0 & 0 & \;\;1 & -0.5\\ 0.25 & 0 & -0.5 & \;\;1 \end{array}]$$

*which allows appreciating the interplay of instantaneous effects with the connectivity/influentiability structures, where the lack of connections/influences measured by iPDC/iDTF is immediately apparent.*

*The computed ${\tilde{\sigma }}_{i}$ are contained in [1.04,1.00,1.17,1.21] and*

(30) $$\tilde{R}=[\begin{array}{rrrr} 1.00 & 0.00 & -0.15 & -0.29\\ 0.00 & 1.00 & 0.00 & 0.00\\ -0.15 & 0.00 & 1.00 & 0.52\\ -0.29 & 0.00 & 0.52 & 1.00 \end{array}]$$

*rounded to two decimal places.*

**Figure 2:**
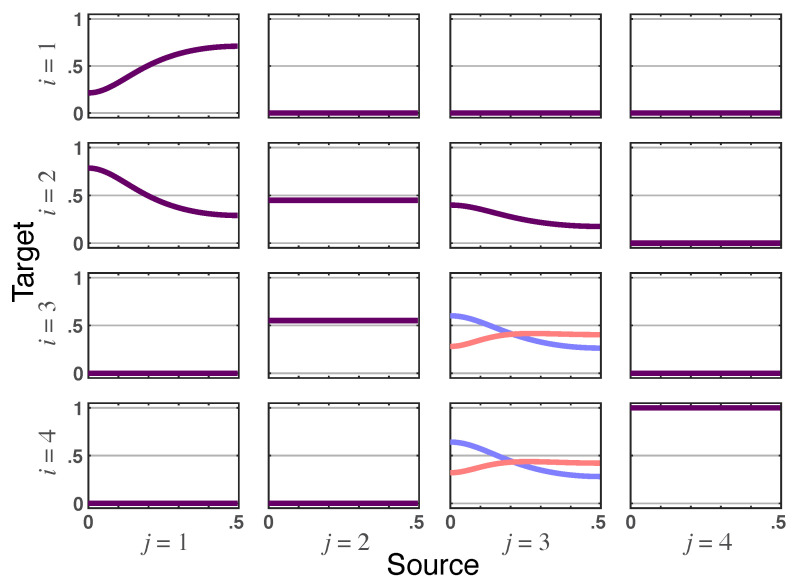
**Squared iPDC**—$|\pi_{ij}{\left(\nu \right)|}^{2}$ (blue lines) and **total PDC magnitude**—$|\overset{⏜}{\pi_{ij}}\left(\nu \right)|$ (red lines) depicted for Example 1. When identical, the superposed traces are shown as dark purple lines.

*The various quantities are represented in the allied graphs showing that iPDC instantaneous effects require the joint presence of partial correlations in $\tilde{R}$ and the presence of immediate connections (see Figure 2). This conjunction only occurs from $x_{3}\left(n\right)$ to $x_{4}\left(n\right)$.*

**Figure 3:**
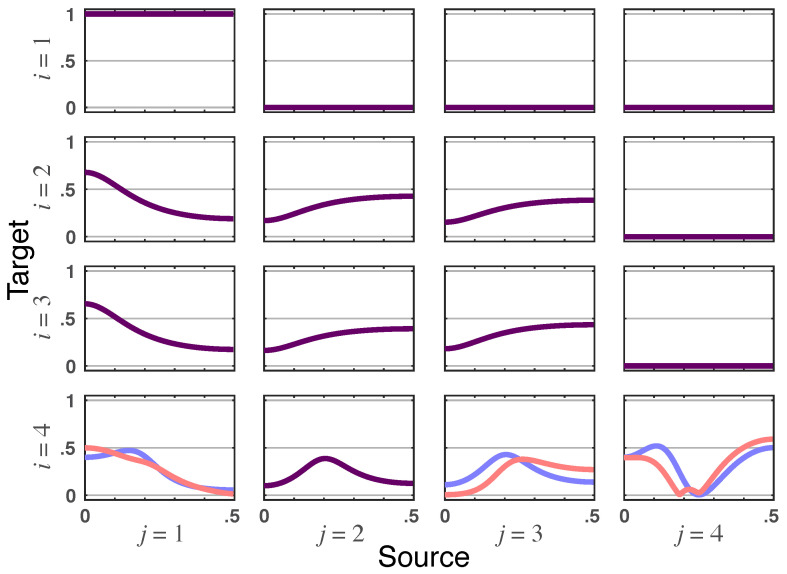
**Squared iDTF**—$|\gamma_{ij}{\left(\nu \right)|}^{2}$ (blue lines) and **total DTF magnitude**—$|\overset{⏜}{\gamma_{ij}}\left(\nu \right)|$ (red lines) rendered for Example 1. When identical, the superposed graphs are shown as dark purple lines.

*Likewise, iDTF instantaneous impacts require the existence of correlations in R so that altered influentiability occurs from $x_{1}\left(n\right)$ to $x_{4}\left(n\right)$ but not in the opposite direction. Something similar also takes place when $x_{3}\left(n\right)$ toward $x_{4}\left(n\right)$ is examined but not in the reverse direction (see Figure 3).*

**Example** **2.** *To provide a clearer idea of iGC frequency domain repercussions for the same time domain characterization as summarized by*

(31) $$\Sigma_{w}=[\begin{array}{cc} 1 & 0.5\\ 0.5 & 1.25 \end{array}]$$

*we consider a set of four slightly different bivariate systems.*

***Example*** *2.1* ***Disconnected System***

*Let the simplest one be described by*

(32) $$A_{1}=\left[\begin{array}{cc} 1.3859 & 0\\ 0 & 0.5 \end{array}\right]$$

*and*

(33) $$A_{2}=\left[\begin{array}{cc} -0.9604 & 0\\ \;0 & 0 \end{array}\right]$$

**Figure 4:**
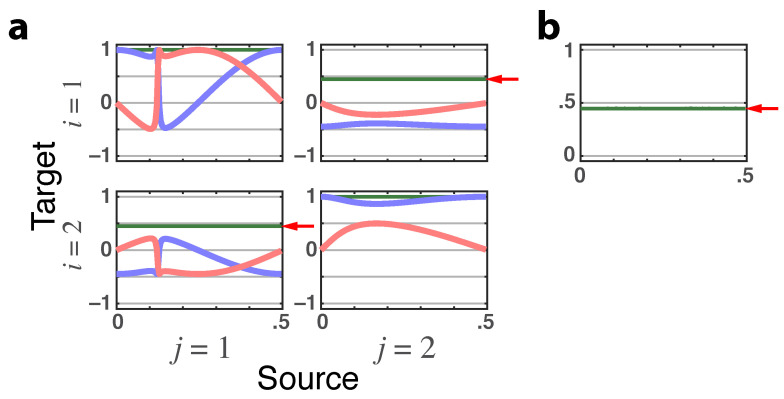
(**a**) **Latent directed instantaneous connectivity**—${\tilde{\pi }}_{ij}\left(\nu \right)$**magnitude** depicted as green lines, shown with its **real** (blue lines) and **imaginary** (red lines) parts that portray iGC effects before G–C inclusion. The value 0.447 of $|{\tilde{\pi }}_{ij}\left(\nu \right)|$, for i≠j, (red arrows) is the same as in **b**. (**b**) **Cross-coherence magnitude**$|C_{12}\left(\nu \right)|=|C_{21}\left(\nu \right)|=0.447=|{\tilde{\pi }}_{12}\left(\nu \right)|=|{\tilde{\pi }}_{21}\left(\nu \right)|$ as the red arrow indicates (Example 2.1).

*The observed total DTF/PDC are trivially equal to zero for i≠j, yet because of iGC as represented by (31), one sees that it manifests itself through a constant $|{\tilde{\gamma }}_{ij}\left(\nu \right)|=|{\tilde{\pi }}_{ij}\left(\nu \right)|=0.447$ that, in turn, leads to a constant magnitude coherence $|C_{12}\left(\nu \right)|$ of the same value as indicated by red arrows on Figure 4a,b.*

*In fact, it is possible to show that absence of G-connectivity implies $|C_{12}\left(\nu \right)|$ is constant. The converse, however, is not generally valid. The results are in accord with the absence of delayed effects between channels (no Granger causality).*

***Example*** *2.2* ***Unidirectional Granger Causality***

*If (32) is replaced by*

(34) $$A_{1}=\left[\begin{array}{cc} 1.3859 & 0\\ 0.5 & 0.5 \end{array}\right]$$

**Figure 5:**
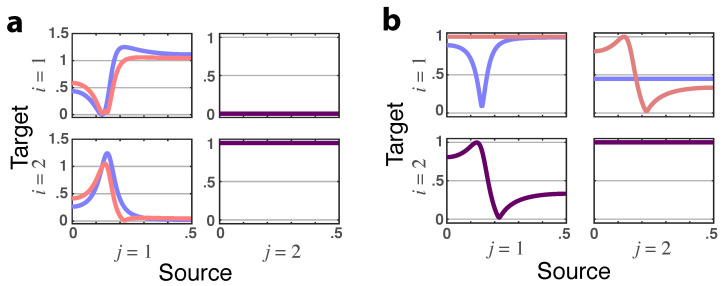
(**a**) **Squared iPDC**—$|\pi_{ij}{\left(\nu \right)|}^{2}$ (blue lines) and **total PDC magnitude**—$|\overset{⏜}{\pi_{ij}}\left(\nu \right)|$ (red lines) for Example 2.2 portraying the absent feedback from $x_{2}\left(n\right)\to x_{1}\left(n\right)$. When identical, the superposed traces are shown as dark purple lines. (**b**) **Latent directed instantaneous connectivity magnitude**—$|{\tilde{\pi }}_{ij}\left(\nu \right)|$ (blue lines) and **partial coherence magnitude**—$|\kappa_{ij}\left(\nu \right)|$ (redlines), which show up as dark purple when traces are identical, for Example 2.2.

*we obtain a total PDC that reflects this change and still detects the lack of $x_{2}\left(n\right)\to x_{1}\left(n\right)$ feedback (Figure 5a). Furthermore, comparing $|{\tilde{\pi }}_{21}\left(\nu \right)|$ to $|\kappa_{ij}\left(\nu \right)|$ in Figure 5b, we see that the unidirectional effect of $x_{1}\left(n\right)$ over $x_{2}\left(n\right)$ is what solely determines the magnitude of the resulting partial coherence.*

***Example*** *2.3* ***Instantaneous link between $x_{1}\left(n\right)$ and $x_{2}\left(n\right)$.***

*Now consider the data generation model given by*

(35) $$\begin{array}{ccc} x_{1}\left(n\right) & = & 1.3859x_{1}\left(n-1\right)-0.9604x_{1}\left(n-2\right)+\epsilon_{1}\left(n\right) \end{array}$$

(36) $$\begin{array}{ccc} x_{2}\left(n\right) & = & 0.5x_{1}\left(n\right)+0.5x_{2}\left(n-1\right)+\epsilon_{2}\left(n\right) \end{array}$$

*where $\epsilon_{i}\left(n\right)$ are independent identically distributed zero mean innovation processes.*

*Under least squares estimation, (1) ideally results in the model given by*

(37) $$A_{1}=\left[\begin{array}{cc} 1.3859 & 0\\ 0.693 & 0.5 \end{array}\right]$$

*and*

(38) $$A_{2}=\left[\begin{array}{cc} -0.9604 & 0\\ -0.4802 & 0 \end{array}\right]$$

*whose residual covariance matrix is also given by (31). This is easy to show by inserting (35) into (36).*

**Figure 6:**
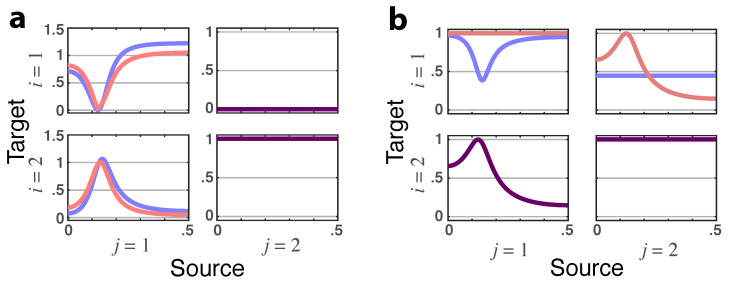
(**a**) **Squared iPDC**—$|\pi_{ij}{\left(\nu \right)|}^{2}$ (blue lines) and **total PDC magnitude**—$|\overset{⏜}{\pi_{ij}}\left(\nu \right)|$ (red lines) plots for Example 2.3 portraying the absent feedback from $x_{2}\left(n\right)\to x_{1}\left(n\right)$. (**b**) **Latent directed instantaneous connectivity magnitude**—$|{\tilde{\pi }}_{ij}\left(\nu \right)|$ (blue lines) and **partial coherence magnitude**—$|\kappa_{ij}\left(\nu \right)|$ (red lines) for Example 2.3, which show up as dark purple when traces coincide.

**Figure 7:**
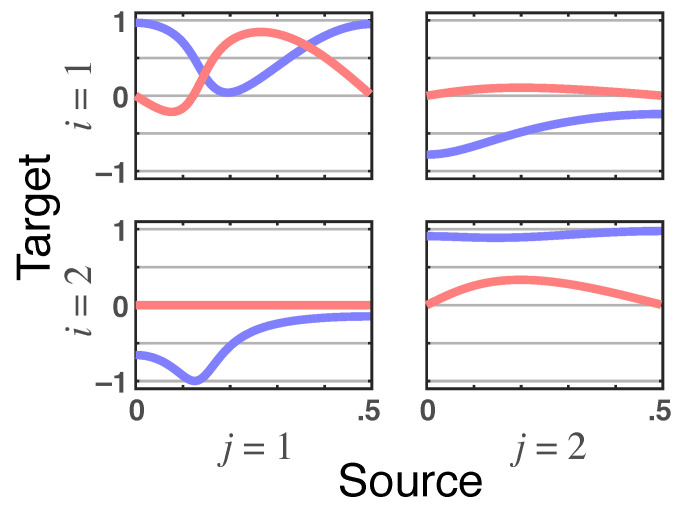
**Latent directed instantaneous connectivity**—${\tilde{\pi }}_{ij}\left(\nu \right)$’s **real** (blue lines) and **imaginary** (red lines) parts plots for Example 2.3. Note that ${\tilde{\pi }}_{21}\left(\nu \right)$’s imaginary part nullity is a signature of their delayless relationship.

*The resulting total PDC is shown in Figure 6a (red lines) whereas the magnitude of $|{\tilde{\pi }}_{ij}\left(\nu \right)|$ (Figure 6b) is further broken into its real and imaginary parts in Figure 7, where again the nullity of the imaginary part of ${\tilde{\pi }}_{21}\left(\nu \right)$ constitutes a signature of the delayless relationship between $x_{1}\left(n\right)$ and $x_{2}\left(n\right)$.*

*Again, because iPDC from $x_{2}\left(n\right)$ to $x_{1}\left(n\right)$ is zero, the partial coherence magnitude $|\kappa_{ij}\left(\nu \right)|=|{\tilde{\pi }}_{21}\left(\nu \right)|$ (i≠j).*

***Example*** *2.4* ***Bidirectional Feedback***

**Figure 8:**
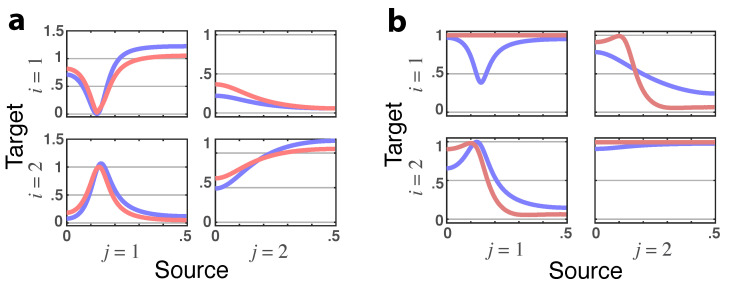
(**a**) **Squared iPDC**—$|\pi_{ij}{\left(\nu \right)|}^{2}$ (blue lines) and **total PDC magnitude**—$|\overset{⏜}{\pi_{ij}}\left(\nu \right)|$ (red lines) plots for Example 2.4. (**b**) Example 2.4’s **latent directed instantaneous connectivity magnitude**—$|{\tilde{\pi }}_{ij}\left(\nu \right)|$ (blue lines) and **partial coherence magnitude**—$|\kappa_{ij}\left(\nu \right)|$ (red lines) plots with no dark purple coincidence line.

**Figure 9:**
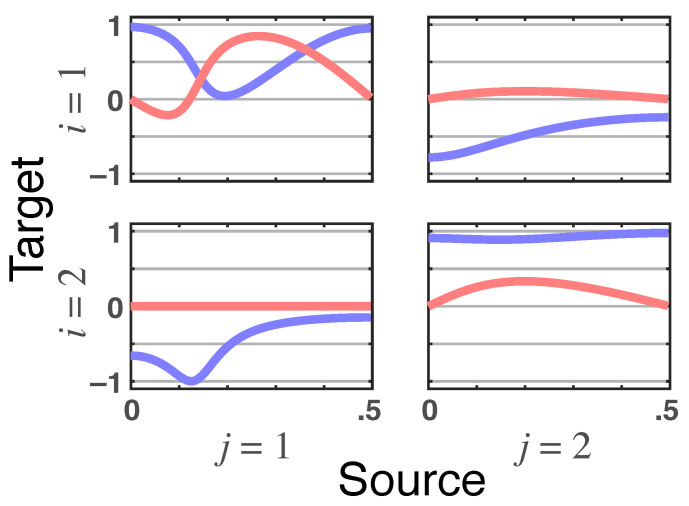
Example 2.4’s **latent directed instantaneous connectivity**—${\tilde{\pi }}_{ij}\left(\nu \right)$**real** (blue) and **imaginary** (red) parts. Note that ${\tilde{\pi }}_{21}\left(\nu \right)$’s imaginary part nullity is a signature of their delayless relationship.

*The introduction of a $0.5x_{2}\left(n-1\right)$ feedback into (35) leads to the total PDC in Figure 8a with the allied magnitude ${\tilde{\pi }}_{ij}\left(\nu \right)$—latent directed instantaneous connectivity—in Figure 8b split into its real and imaginary parts in Figure 9 where the delayless $x_{1}\left(n\right)$ to $x_{2}\left(n\right)$ instantaneous description remains unaffected, while the partial coherence $|\kappa_{12}\left(\nu \right)|$ now depends on both directions.*

**Example** **3.** *This example is borrowed from [7] whose theoretically equivalent model (Figure 6.3a in [7]) as obtained by fitting (1) is given by:*

(39) $$A_{1}=\left[\begin{array}{ccc} 1.27 & 0.00 & 0.00\\ 0.64 & 0.00 & 1.00\\ 0.32 & 0.00 & 0.50 \end{array}\right]$$

(40) $$A_{2}=\left[\begin{array}{ccc} -0.81 & 0.00 & 0.00\\ -0.41 & 0.00 & 0.00\\ -0.20 & 0.50 & -0.64 \end{array}\right]$$

*and*

(41) $$\Sigma_{w}=\left[\begin{array}{ccc} 1.000 & 0.500 & 0.250\\ 0.500 & 2.250 & 1.125\\ 0.250 & 1.125 & 3.562 \end{array}\right]$$

*which leads to $\tilde{\sigma }=\left[1.06,1.15,1.09\right]$ and*

(42) $$\tilde{R}=\left[\begin{array}{rrr} \;1.00 & -0.31 & \;0.00\\ -0.31 & 1.00 & -0.38\\ \;0.00 & -0.38 & \;1.00 \end{array}\right]$$

*rounded to two decimal digits.*

**Figure 10:**
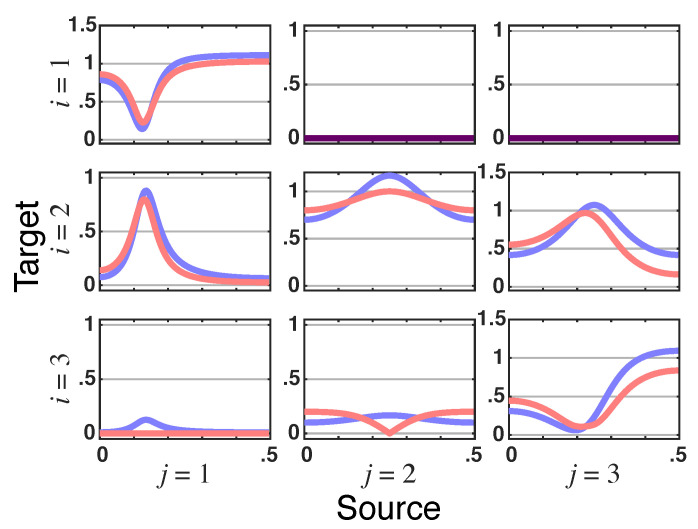
**Squared PDC**—$|\pi_{ij}{\left(\nu \right)|}^{2}$ (blue) and **total PDC magnitude**—$|\overset{⏜}{\pi_{ij}}\left(\nu \right)|$ (red) plots, indicated as dark purple lines when traces coincide, from Example 3. Observe that $|\overset{⏜}{\pi_{31}}\left(\nu \right)|=0$ points to a lack of total G-connectivity from $x_{1}\left(n\right)$ to $x_{3}\left(n\right)$.

**Figure 11:**
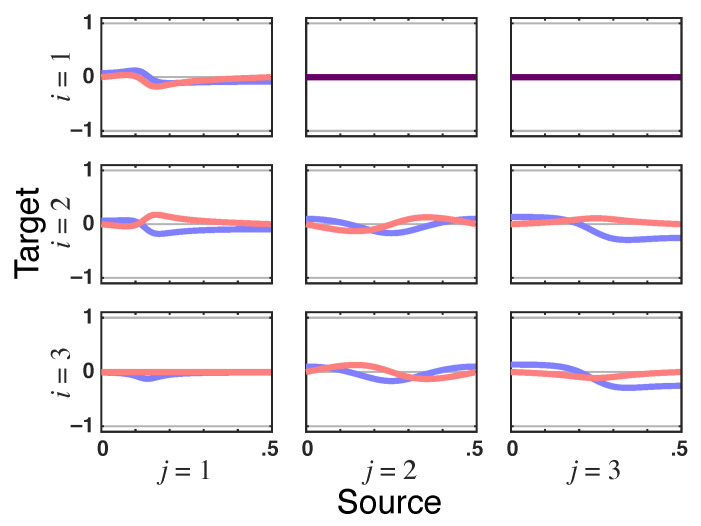
**Residual directed PDC**—${\overset{﹀}{\pi }}_{ij}\left(\nu \right)$’s **real** (blue lines) and **imaginary** (red lines) parts plots in Example 3, which show up as dark purple when traces coincide. Note ${\overset{﹀}{\pi }}_{31}\left(\nu \right)$’s imaginary part nullity, which is a signature of their delayless relationship but whose real part is equal and of the opposite sign to squared iPDC ($|\pi_{31}{\left(\nu \right)|}^{2}$), thereby leading to zero total PDC from $x_{1}\left(n\right)$ to $x_{3}\left(n\right)$.

*What stands out is that total PDC is identically zero for $x_{1}\left(n\right)\to x_{3}\left(n\right)$ due to the presence of instantaneous Granger interactions (Figure 10). This nullity is consistent with the structure inferred in [7] when instantaneous quantities are considered by including a zero term lag in (1) (Figure 6.3b in [7]). This happens because ${\overset{﹀}{\pi }}_{31}\left(\nu \right)$—the residual directed PDC from $x_{1}\left(n\right)\to x_{3}\left(n\right)$—in (27) is of the opposite sign and instantaneously undoes the effect of iPDC ($|\pi_{31}\left(\nu \right){|}^{2})$ as it too has no delay (look at the $x_{1}\left(n\right)\to x_{3}\left(n\right)$ panel in Figure 11).*

**Figure 12:**
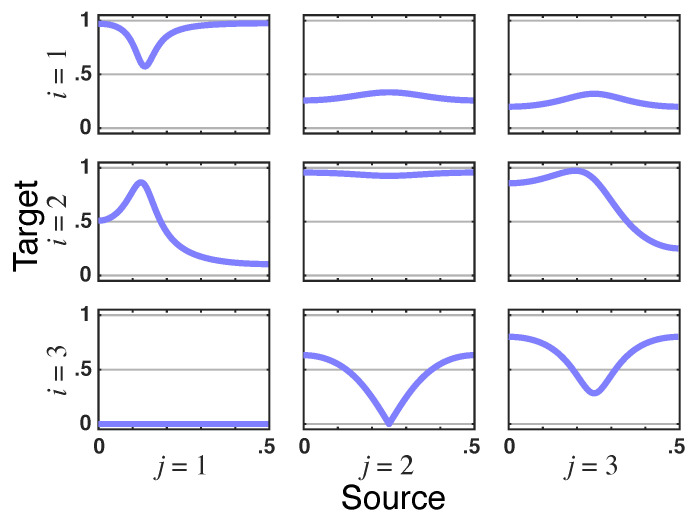
**Latent directed instantaneous connectivity magnitude**—$|{\tilde{\pi }}_{ij}\left(\nu \right)|$ for Example 3, where it is to $|{\tilde{\pi }}_{31}\left(\nu \right)|=0$ such that the corresponding total PDC is zero.

**Figure 13:**
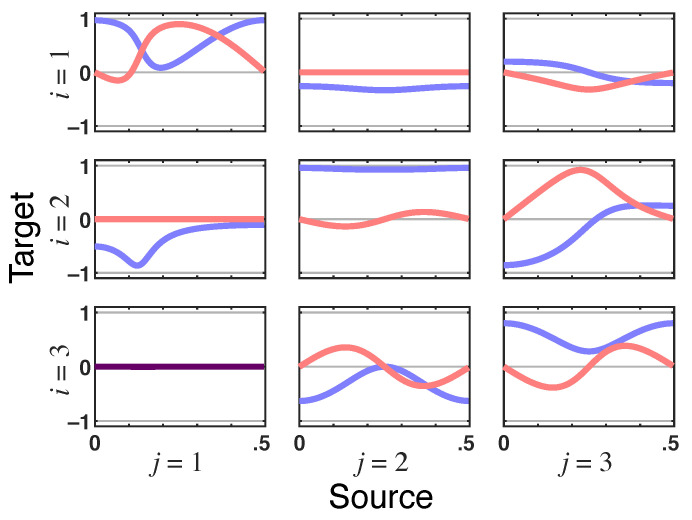
**Latent directed instantaneous connectivity**—${\tilde{\pi }}_{ij}\left(\nu \right)$**real** (blue lines) and **imaginary** (red lines) parts plots for Example 3. Note that ${\tilde{\pi }}_{21}\left(\nu \right)$ and ${\tilde{\pi }}_{31}\left(\nu \right)$ have zero imaginary parts that point to delayless directed relationships in this example. The real part of ${\tilde{\pi }}_{31}\left(\nu \right)$ is also zero (depicted as dark purple line).

*Since the relationship of $x_{1}\left(n\right)$ to $x_{2}\left(n\right)$ is also instantaneous as portrayed by the nullity of the imaginary part of ${\tilde{\pi }}_{21}\left(\nu \right)$, it is clear that $x_{2}\left(n\right)$ mediates this total PDC nullity from $x_{1}\left(n\right)$ to $x_{3}\left(n\right)$. Note as well that $|{\tilde{\pi }}_{31}\left(\nu \right)|=0$ (Figure 12). The instantaneous link from $x_{1}\left(n\right)$ to $x_{2}\left(n\right)$ is apparent in the nullity of the imaginary part of ${\tilde{\pi }}_{21}\left(\nu \right)$ in Figure 13; note also the same nullity in ${\tilde{\pi }}_{21}\left(\nu \right)$, whose real part is also zero consistently with zero total PDC from $x_{1}\left(n\right)$ to $x_{3}\left(n\right)$.*

*Together, these observations lead to the conclusion that the relationship from $x_{1}\left(n\right)$ to both $x_{2}\left(n\right)$ and $x_{3}\left(n\right)$ are instantaneous and mediated without delay, and that the one from $x_{1}\left(n\right)$ must occur through $x_{2}\left(n\right)$ since the total PDC from it to $x_{3}\left(n\right)$ is zero.*

## 5. Discussion

The present expanded formulation takes care of the problem frequently met in data analysis whose residuals in fitting (1) result in being mutually correlated and its consequences.

By examining the decomposition of coherencies and partial coherencies in terms of the information versions of DTF and PDC [9], we managed several things that lend the latter quantities a fundamental theoretical character.

The first such result was to show that the allied properly generalized total quantities enjoy the same kind of normalization as the original DTF/PDC [3,4] (DC/gPDC [5,13]). Likewise, the same ’inversion’ properties of the latter hold for the former when N=2. A key point in obtaining the present symmetry of treatment between DTF and PDC was iPDC’s rescaling (15).

The second result is that of emphasizing the importance of the magnitude squared iDTF/iPDC in portraying, respectively, G-influentiability and G-connectivity that now allow an extended picture to be drawn: that of Granger instantaneous influentiability (G–iI) and connectivity (G–iC) by now considering the total DTF and total PDC, which are also directed quantities.

One important aspect as portrayed in Example 1 is that instantaneous directedness effects are due to the combined effect of non-zero off-diagonal $\Sigma_{w}$ terms and non-zero iDTF/iPDC.

Likewise the role of latent instantaneous iPDC (21) permits the careful analysis of instances of instantaneous interaction as illustrated in Example 2.3 and Example 3.

Through Example 2, we learned that the very same time domain description of instantaneous Granger causality has quite a few distinct repercussions depending on the underlying G-connectivity that can only be properly described in the frequency domain.

When compared to other Granger dynamical characterizations that include instantaneous considerations, the present formulation has the advantage of dispensing with special model estimation approaches. No special model to include the r=0 lag in (1) is required with its more elaborate estimation considerations [7]. Likewise, also unneeded are the estimations of multiple models as in [8]. All that is required is a standard least squares model adjustment via (1), wherefrom all conclusions can be drawn.

There is still much work ahead. Here, to keep focus, we have exclusively examined the details of $C_{ij}\left(\nu \right)$ and $\kappa_{ij}\left(\nu \right)$; when i=j, our next step is to examine the more general i≠j case. Also needed now is the establishment of detailed asymptotic results for the newly introduced total quantities as are available for iDTF [14] and iPDC [15].

## 6. Conclusions

The present formulation has developed the necessary formalism to address the repercussions of instantaneous Granger causality, whose proper description demands the frequency domain, where they were shown to be dependent on G-connectivity details for size and directedness. Also confirmed is our statement that iDTF/iPDC are natural fundamental quantities that result from the respective decomposition of coherency and partial coherency.

## Figures and Tables

**Table 1:** Symbol definitions for the quantities in the text. Equation numbers where they first appear are also shown.

Quantity	Matrix	Elements	Equation
iDTF	Γ(ν)	$\gamma_{ij}\left(\nu \right)$	(7)
Latent directed instantaneous DTF	Γ(ν)R	${\tilde{\gamma }}_{ij}\left(\nu \right)$	(20)
Squared iDTF	$\Gamma \left(\nu \right)⊙\Gamma^{*}\;\left(\nu \right)$	$|\gamma_{ij}{\left(\nu \right)|}^{2}$	(25)
Total DTF (tDTF)	$\overset{⏜}{\;\;\Gamma \;\;}\;(\nu )$	$\overset{⏜}{\;\;\gamma_{ij}\;\;}\left(\nu \right)$	(25)
Residual directed DTF	$\Gamma \left(\nu \right)\rho ⊙\Gamma^{*}\;\left(\nu \right)$	$\gamma_{ij}\left(\nu \right)$	(25)
iPDC	Π(ν)	$\pi_{ij}\left(\nu \right)$	(14,15) (see text)
Latent directed instantaneous PDC	$\tilde{R}\Pi \left(\nu \right)$	${\tilde{\pi }}_{ij}\left(\nu \right)$	(21)
Squared iPDC	$\Pi^{*}\;\left(\nu \right)⊙\Pi \left(\nu \right)$	$|\pi_{ij}{\left(\nu \right)|}^{2}$	(27)
Total PDC (tPDC)	$\overset{⏜}{\;\;\Pi \;\;}\;(\nu )$	$\overset{⏜}{\;\;\pi_{ij}\;\;}\left(\nu \right)$	(27)
Residual directed PDC	$\Pi^{*}\;\left(\nu \right)⊙\tilde{\rho }\Pi \left(\nu \right)$	$\pi_{ij}\left(\nu \right)$	(27)

## Data Availability

Data sharing not applicable.

## Abbreviations

The following abbreviations are used in this manuscript:

iGC	Instantaneous Granger causality
G–C	Granger connectivity
G–I	Granger influentiability
G–iI	Granger instantaneous influentiability
G–iC	Granger instantaneous connectivity
DTF	Directed transfer function
DC	Directed coherence
iDTF	Information directed transfer function
tDTF	Total directed transfer function
PDC	Partial directed coherence
gPDC	Generalized partial directed coherence
iPDC	Information partial directed coherence
tPDC	Total partial directed coherence
